# Effect of anti-epileptic drugs on the survival of patients with glioblastoma multiforme: A retrospective, single-center study

**DOI:** 10.1371/journal.pone.0225599

**Published:** 2019-12-02

**Authors:** Jae Yeoul Ryu, Kyoung Lok Min, Min Jung Chang

**Affiliations:** 1 Department of Pharmacy and Yonsei Institute of Pharmaceutical Sciences, College of Pharmacy, Yonsei University, Incheon, Republic of Korea; 2 Department of Pharmaceutical Medicines and Regulatory Science, Colleges of Medicine and Pharmacy, Yonsei University, Incheon, Republic of Korea; Goethe University Hospital Frankfurt, GERMANY

## Abstract

Glioblastoma multiforme (GBM) is a lethal and aggressive malignant tumor of the central nervous system. The World Health Organization classifies it as a grade IV astrocytoma. Controlling seizures is essential during GBM treatment because they are often present and closely associated with the quality of life of GBM patients. Some antiepileptic drugs (AEDs) exhibit antitumor effects and could decrease the mortality of patients with GBM. In this retrospective cohort study, we examined 418 patients treated with surgery, radiotherapy, and chemotherapy with temozolomide (TMZ) at Severance Hospital in South Korea, per the current protocol. Median overall survival (OS) was 21 months [95% confidence interval (CI): 18.1–23.9] in the levetiracetam (LEV) treatment group, whereas it was 16 months [95% CI: 14.1–17.9] in the group without LEV, exhibiting a statistically significant difference between the two groups (*P* < 0.001). Of nine AED groups, only LEV treatment [*P* = 0.001; hazard ratio (HR), 0.65; 95% CI: 0.51–0.83] exhibited a statistically significant difference in the OS, in the univariate analysis. In the risk analysis of the baseline characteristics, age, administration of LEV, and O6-methylguanine-DNA methyltransferase (MGMT) promoter status correlated with OS. The use of LEV in the group with a methylated MGMT promoter resulted in a positive impact on the OS [*P* = 0.006; HR, 0.174; 95% CI: 0.050–0.608], but the effect of LEV on the OS was not statistically significant in the unmethylated MGMT promoter group (*P* = 0.623). This study suggests that, compared with other AEDs, the administration of LEV may prolong the survival period in GBM patients with methylated MGMT promoters, who are undergoing chemotherapy with TMZ.

## Introduction

Glioblastoma multiforme (GBM) is a lethal and aggressive malignant tumor of the central nervous system. GBM is characterized by nuclear atypia, astrocytic differentiation, cellular polymorphism, microvascular proliferation, and necrosis [1]. In 2013, the Central Brain Tumor Registry of the United States reported the incidence rate of GBM as only 3.19 per 100,000 [2]. However, <25% of patients with GBM survive >2 years without disease progression, despite adhering to protocol-based procedures for brain tumors [3,4]. The European Society for Medical Oncology (ESMO) recommends concomitant chemoradiotherapy (CCRT), radiotherapy for 5 days a week for 6 weeks along with daily TMZ (dose: 75 mg/m^2^) and, then, maintenance TMZ (dose: 150–200 mg/m^2^ dose) implemented daily for 5 days every 28 days for 6–12 cycles [5–7].

The prognosis of patients with GBM can be predicted using glioma-related genetic factors. The Ki-67 protein can be found in proliferating cells and is responsible for the induction and recurrence of glioblastoma. In one study, patients with GBM harboring isocitrate dehydrogenase I (*IDH1*) gene mutations exhibited higher survival rates than patients with GBM harboring the wild-type gene, because glioma cells harboring the mutation exhibit lower enzymatic activity than those with the wild-type gene [8]. In particular, the O6-methylguanine-DNA methyltransferase (*MGMT*) gene is strongly associated with DNA repair. Cancer patients with a methylated MGMT promoter exhibit prolonged survival in comparison with patients harboring an unmethylated MGMT promoter when treated with alkylating agents such as TMZ, which is currently considered the first-line anticancer agent for GBM. In other words, the MGMT promoter status could be an indicator to predict the prognosis and pathogenesis of patients with GBM [5,9]. Reportedly, the presence of the X-linked alpha-thalassemia mental retardation syndrome (ATRX), p53 inhibition, intact 1p/19q, epidermal growth factor receptor (EGFR) amplification, and histone deacetylase (HDAc) expression correlate with poor prognosis and outcomes in patients with GBM [8–12].

Reportedly, seizures are a common symptom, generally presenting in up to 40–45% of patients with GBM [13]. The presence of seizures depends on the genetic mutation, location of the brain tumor, and type or grade of tumor [13]. Controlling seizures is essential during GBM treatment because they are often present and closely associated with the quality of life of patients [14]. Some antiepileptic drugs (AEDs) such as valproic acid (VPA) and levetiracetam (LEV) have exhibited mechanisms to enhance the effectiveness of some antitumor agents and may lead to decreased mortality of patients with GBM [15–17]. Hence, patients with GBM need to be treated with appropriate medications for two reasons, controlling seizures and reducing tumor progression.

Among AEDs, VPA exhibits antitumor effects via HDAc inhibition and modulation of the mitogen-activated protein kinase pathway. In addition, owing to the radio-sensitizing properties of VPA, radiotherapy with VPA is more effective than that with other or no AEDs [18,19]. Furthermore, many recent preclinical studies have suggested that GBM patients treated with VPA exhibit better outcomes than those treated with other AEDs [20–23].

LEV, a relatively new AED, exhibits not only excellent anti-seizure effects but also fewer side effects [24]. A recent study reported that the mechanism underlying the effects of LEV involves the inhibition of *MGMT* gene expression via formation of a complex with p53; moreover, it acts as a sensitizer for TMZ [11]. Some studies have suggested that LEV could be used as first-line medication in patients with brain tumors to control seizures and enhance the effectiveness of chemotherapy, especially high-dose TMZ [25–27].

However, the reported effects of AEDs in patients with GBM remain debatable; moreover, limited research has been conducted on Korean subjects in this regard. Hence, this study aimed to determine the most appropriate therapeutic measures by survival analysis to elucidate the effects of AEDs in patients with GBM in Korea.

## Materials and methods

### Study design

This retrospective, single-center study was conducted at Severance Hospital in Seoul, South Korea. Between January 2005 and May 2017, 628 consecutive patients were diagnosed with histologically confirmed primary GBM; all patient information was gathered from the electronic medical records. The study protocol was approved by the Institutional Review Board (IRB) of the Yonsei University Health System (Seoul, South Korea; IRB No. 4-2017-0659) and all data provided from hospital were anonymized and informed consent was waived by IRB.

The primary outcome related to efficacy was overall survival (OS), which was defined as the time to death, regardless of the cause. Here, the OS with LEV and VPA treatment was compared because both are preferentially recommended in the guideline as they are non-enzyme-inducing agents and less likely to interact with other drugs, including TMZ [6,28]. Further, the OS was compared in the following four ways: (1) patients with AEDs including LEV (LEV group), versus those with AEDs not including LEV (no LEV group); (2) patients with AEDs including VPA (VPA group), versus those with AEDs not including VPA (no VPA group); (3) patients with AEDs including both LEV and VPA (both group), versus the LEV group or the VPA group; and (4) patients receiving only LEV and VPA without other AEDs (only both group) versus only LEV without other AEDs (only LEV group) or only VPA without other AEDs (only VPA group).

We assessed the baseline characteristics, including sex, age, the number of AEDs, whether to use LEV, and genetic factors (IDH1, EGFR, p53, Ki-67 cutoff 10% and 20%, MGMT, 1p, 19q, and ATRX) to adjust for risk or confounding factors. In this study, age and the number of AEDs were considered continuous variables, whereas sex, whether to use LEV, and genetic factors were categorical variables. If a patient was alive at the time of the last follow-up, it was treated as censored data, which were not excluded and the data before the last follow-up were used.

In this study, the inclusion criteria were as follows: patients diagnosed with primary GBM treated with surgery, radiotherapy, and chemotherapy per the protocol; patients who used at least one AED after the diagnosis of primary GBM; and patients with more than three months follow-up. Conversely, the exclusion criteria were as follows: inadequate data in the electronic medical records and patients whose survival or death could not be established. If there were records of the loss of eligibility for a health insurance holder, this date was considered the time of death.

### Treatment protocol

The ESMO recommends CCRT, fractionated radiotherapy (dose: 1.8–2 Gy) for 5 days a week for 6 weeks, which is equivalent to 60 Gy, along with a daily TMZ dose at 75 mg/m^2^, followed by a daily maintenance TMZ dose of 150–200 mg/m^2^ for 5 days every 28 days for 6–12 cycles [5–7].

Notably, AEDs can be used in patients with GBM presenting with seizure symptoms. Reportedly, when using AEDs for seizure prophylaxis, AEDs can be administered before and after surgery [6]. When seizures are not controlled by monotherapy, addition of a secondary AED, rather than monotherapy with high dose, is recommended [26].

### Statistical analysis

We performed the Kaplan–Meier survival analysis for some representative AEDs to estimate the OS differences among patients with GBM who were undergoing treatment with different AEDs; hazard ratio (HR) and 95% confidence intervals (CI) were determined. Additionally, a log-rank test was performed to identify differences among the groups. We assessed risk factors and statistical errors using the univariate Cox proportional hazards model. In the univariate analysis, if *P* < 0.05, each corresponding variable was considered OS-dependent and additional multivariate analysis was performed to eliminate disturbance between variables. All statistical analyses in this study were performed using IBM SPSS^®^ ver. 24 statistical software program (SPSS Inc., Chicago, IL).

## Results

Of 628 consecutive patients diagnosed with GBM, 31 patients who had a follow-up at less than 3 months and 179 patients who did not receive either surgery, radiotherapy, or chemotherapy with TMZ were excluded. Thus, 418 patients were included in the study.

Table 1 summarizes the baseline characteristics of this study cohort. Of 418 patients, 230 (55%) were men and 188 (45%) were women. The median age at diagnosis was 57 years (range: 2–82). The median number of AEDs used in the patients with GBM was 2 (range: 1–9). Regardless of monotherapy or polytherapy of AEDs, 322 patients were treated with LEV, 218 with VPA, 83 with gabapentin, 76 with topiramate, 50 with phenytoin or fosphenytoin, 40 with lamotrigine, 39 with oxcarbazepine, 31 with pregabalin, 14 with carbamazepine, 3 with clobazam, 3 with phenobarbital, 1 with lacosamide, and 1 with perampanel; no patients lacked AED treatment (Table 2).

**Table 1:** The baseline clinical characteristics of patients.

Parameter	No. of patients (%) or Median [Range]
**Total**	418 (100)
**Sex**	
Men	230 (55)
Women	188 (45)
**Age at diagnosis (years)**	57 [2–82]
1–29	30 (7)
30–59	207 (50)
≥60	181 (43)
**Genetic factors**	
IDH1	
Wild-type	216 (52)
Mutation	14 (3)
Missing	188 (45)
EGFR	
Wild-type	71 (17)
Amplification	154 (37)
Missing	193 (46)
p53	
Negative	50 (12)
Positive	176 (42)
Missing	192 (46)
Ki-67	
<10%	46 (11)
10%–19%	74 (18)
≥20%	119 (28)
Missing	179 (43)
MGMT	
Methylated	70 (17)
Unmethylated	155 (37)
Missing	193 (46)
1p/19q	
Intact	171 (41) / 168 (40)
LOH	37 (9) / 40 (10)
Missing	210 (50)
**Survival duration (months)**	19 [3–137]
**The number of AEDs**	2 [1–9]

IDH1, isocitrate dehydrogenase I; EGFR, epidermal growth factor receptor; MGMT, O6-methylguanine-DNA methyltransferase; LOH, loss of heterozygosity; AED, antiepileptic drug.

**Table 2:** The number of patients using AEDs.

AEDs	Monotherapy	Polytherapy	Total
Levetiracetam	132	190	322
Valproic acid	35	183	218
Gabapentin	2	81	83
Topiramate	0	76	76
Phenytoin/Fosphenytoin	0	50	50
Lamotrigine	0	40	40
Oxcarbazepine	0	39	39
Pregabalin	1	30	31
Carbamazepine	0	14	14
Clobazam	0	3	3
Phenobarbital	0	3	3
Lacosamide	0	1	1
Perampanel	0	1	1

AED, antiepileptic drug.

None of the 418 patients who received AED exhibited any severe side effects, such as Stevens-Johnson syndrome, anaphylaxis, suicidal ideation, and toxic epidermal necrolysis.

**Fig 1:**
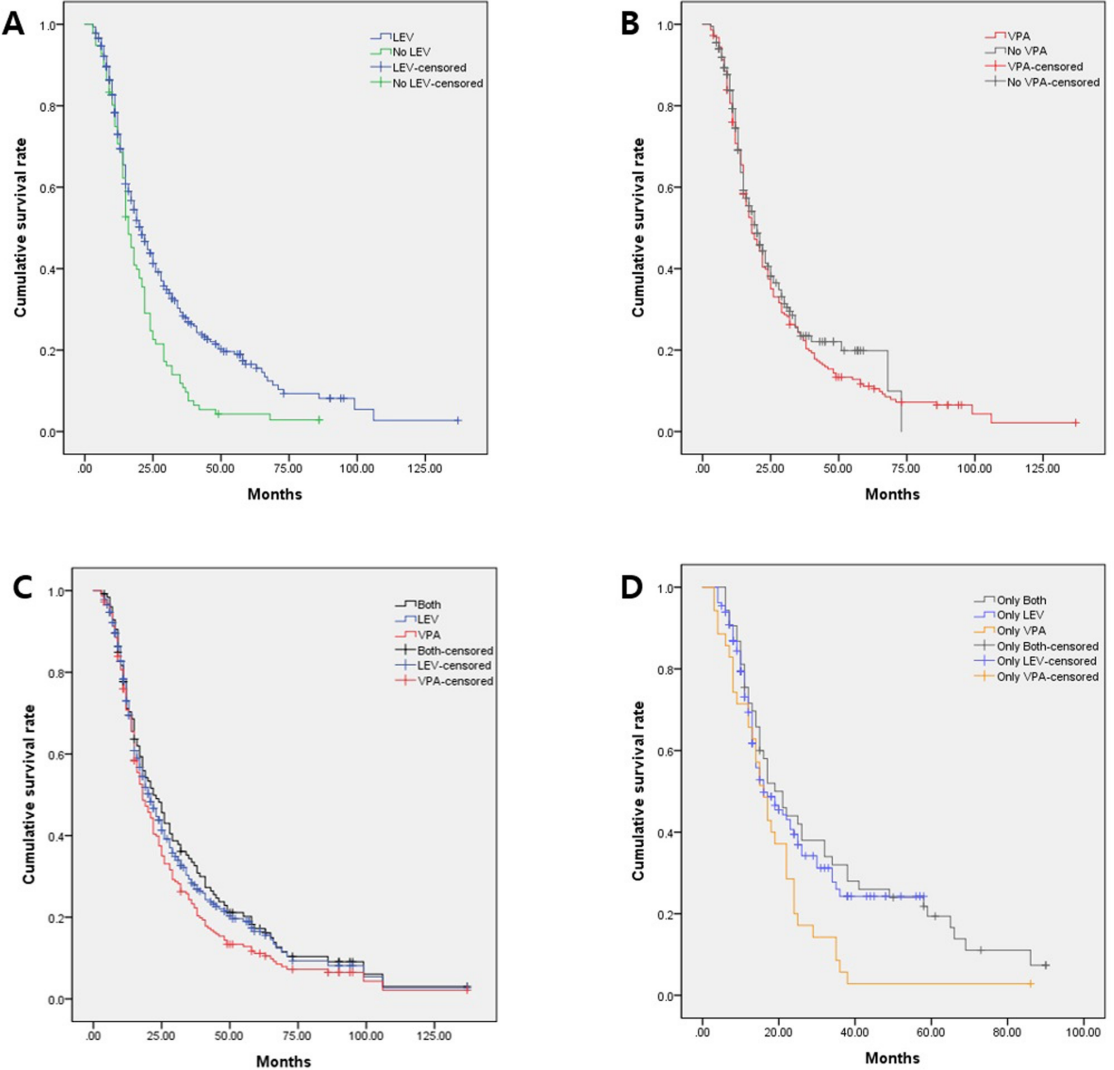
The Cox proportional hazard regression forest plot showing the risk of each antiepileptic drug in patients with glioblastoma. HR, hazard ratio; CI, confidence interval.

We analyzed the effects of AEDs when administered to >10 patients using the Cox proportional model (Fig 1). Of the nine AED groups, only LEV treatment (*P* = 0.001; HR, 0.65; 95% CI: 0.51–0.83) correlated significantly with the OS in the univariate analysis. We observed no positive effects on the OS in the other AED groups.

**Fig 2:**
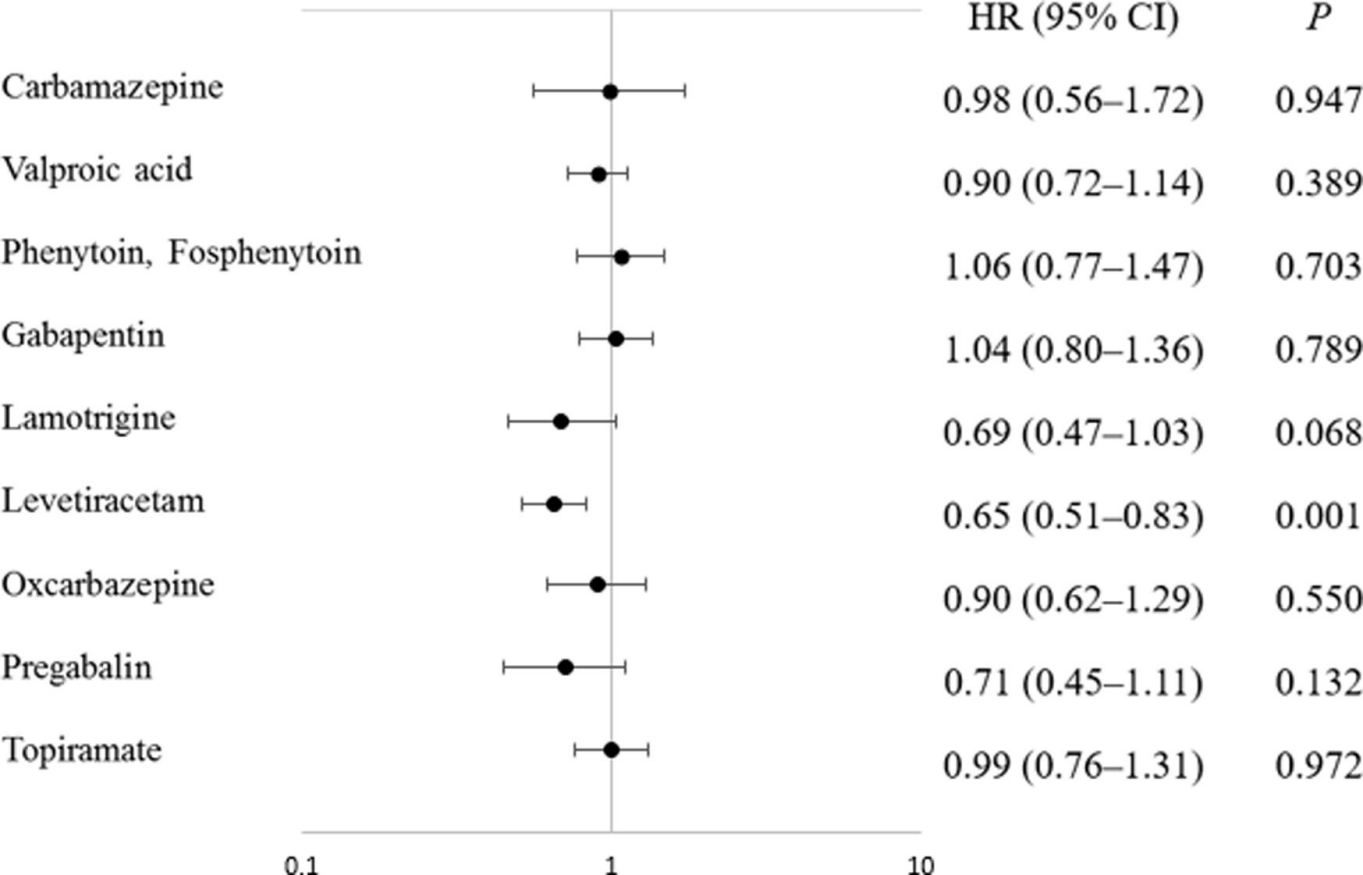
The Kaplan–Meier survival plot showing the overall survival (OS) duration. (a) The LEV-treated group (LEV; blue line) versus no LEV treatment (No LEV; green line). (b) The VPA-treated group (VPA; red line) versus no VPA treatment (No VPA; gray line). (c) The group treated with both LEV and VPA (both; black line) versus the LEV-treated group (blue line) or the VPA-treated group (red line). (d) Treatment with only LEV and VPA (only both; silver line) versus only LEV treatment (only LEV; azure line) or only VPA treatment (only VPA; orange line).

The median OS of all patients was 19 months (range: 3–137; 95% CI: 16.9–21.1). The median OS was 21 months (95% CI: 18.1–23.9) in the LEV group and 16 months (95% CI: 14.1–17.9) in no LEV group with the difference between these groups exhibiting statistical significance (*P* < 0.001; Fig 2A). In addition, the median OS was 18 months (95% CI: 15.0–21.0) in the VPA group and 20 months (95% CI: 16.5–23.5) in the no VPA group, but the difference was not statistically significant (*P* = 0.38; Fig 2B). For both group, the median OS was 22 months (95% CI: 16.5–27.5; Fig 2C). For only LEV or only VPA group, the median OS was 16 months for both, with 95% confidence intervals of 10.3–21.7 and 12.5–19.5, respectively. For only both group, the median OS was 19 months (95% CI: 13.2–24.9; Fig 2D).

In the risk analysis of the baseline characteristics (see Table 3), older age (*P* < 0.001; HR, 1.015; 95% CI: 1.007–1.024), LEV administration (*P* = 0.001; HR, 0.651; 95% CI: 0.510–0.832), an *IDH1* mutation (*P* = 0.014; HR, 0.357; 95% CI: 0.158–0.809), MGMT promoter methylation (*P* < 0.001; HR, 0.428; 95% CI: 0.297–0.616), and a 19q deletion (*P* = 0.032; HR, 0.638; 95% CI: 0.423–0.961) correlated with the OS. The multivariate analysis between these factors revealed that older age (*P* = 0.003; HR, 1.019; 95% CI: 1.006–1.032), LEV administration (*P* = 0.029; HR, 0.393; 95% CI: 0.171–0.907), and MGMT promoter methylation (*P* < 0.001; HR, 0.397; 95% CI: 0.268–0.589) significantly affected the OS. In other words, patients with GBM who were elderly, had a methylated MGMT promoter, and were treated with LEV exhibited longer OS than those who did not have these features.

**Table 3:** Baseline risk factors for the overall survival of patients with glioblastoma.

Factor	Univariate		Multivariate	
	HR (95% CI)	*P*	HR (95% CI)	*P*
Sex (women)	0.994 (0.796–1.241)	0.955		
Age (elderly)	**1.015 (1.007**–**1.024)**	**< 0.001**	**1.019 (1.006**–**1.032)**	**0.003**
Number of AEDs	0.936 (0.858–1.022)	0.139		
Levetiracetam	**0.651 (0.510**–**0.832)**	**0.001**	**0.393 (0.171**–**0.907)**	**0.029**
*IDH1* (mutation)	**0.357 (0.158**–**0.809)**	**0.014**	0.639 (0.257–1.591)	0.336
EGFR	0.984 (0.688–1.407)	0.929		
p53	1.039 (0.726–1.486)	0.834		
Ki-67 (**≥**10%)	1.383 (0.922–2.076)	0.117		
Ki-67 (**≥**20%)	1.029 (0.764–1.388)	0.849		
MGMT (methylation)	**0.428 (0.297–0.616)**	**<0.001**	**0.397 (0.268–0.589)**	**<0.001**
1p	0.846 (0.561–1.276)	0.425		
19q	**0.638 (0.423**–**0.961)**	**0.032**	0.735 (0.484–1.117)	0.149
ATRX	1.023 (0.915–1.144)	0.686		

HR, hazard ratio; CI, confidence interval; AED, antiepileptic drug; IDH1, isocitrate dehydrogenase I; EGFR, epidermal growth factor receptor, MGMT; O6-methylguanine-DNA methyltransferase; ATRX, X-linked alpha-thalassemia mental retardation syndrome.

We conducted a risk analysis of patients with GBM who underwent testing to determine the methylation status of the MGMT promoter by dividing the group as per the methylation status to determine the relevance of LEV treatment and promoter status (Table 4). With MGMT promoter methylation, which is associated with a favorable prognosis, the LEV group displayed a much lower risk to the OS than no LEV group (*P* = 0.006; HR, 0.174; 95% CI: 0.050–0.608). In contrast, in the group with the MGMT promoter unmethylation, we did not observe a significant difference in risk to survival between the LEV group and no LEV group (*P* = 0.623).

**Table 4:** Comparison of survival risks using LEV and MGMT promoter status.

MGMT status	Number of patients		HR (95% CI)	*P*
	LEV	No LEV		
Methylation	66	4	**0.174 (0.050–0.608)**	**0.006**
Unmethylation	148	7	0.810 (0.351–1.874)	0.623

LEV, levetiracetam; MGMT, O6-methylguanine-DNA methyltransferase; HR, hazard ratio; CI, confidence interval.

## Discussion

This study primarily aimed to identify the AED that is the most effective in increasing the OS of patients with GBM. The findings suggest that treatment with LEV was associated with the most improvement in survival compared with VPA, the other nine AEDs available to patients with GBM, irrespective of whether AED monotherapy or polytherapy was utilized. To our knowledge, this study is the first in South Korea to investigate the survival of patients with GBM based on AED use; moreover, this is a practical approach as it is common for patients to receive more than one AED in clinical practice. In this study, 170 patients (41%) received only 1 AED, whereas 248 patients (59%) received more than 2 AEDs.

Fig 1 shows HRs for each AED. Among these, LEV, VPA, and lamotrigine showed lower HRs compared to the other AEDs and only LEV showed statistical significance. In Fig 2A, the LEV group showed a five-month increase in survival compared to no LEV group, but the VPA group showed a two-month decrease in survival compared to no VPA group (Fig 2B). This is a result of the fact that the number of patients using LEV in our cohort was as high as 77%. If one AED is not enough to control symptoms, it is more effective to add another AED rather than replacing it with another AED [26]. About 60% of patients with GBM in our cohort consequently received more than two AEDs, of which LEV was preferred as a primary or secondary medication because of its relatively low side effects and wide therapeutic range [24,26].

In Fig 2D, the “only LEV” group and the “only VPA” group were compared to reduce the disturbance between LEV and VPA. The median OS of the “only both group” was 22 months, whereas the median OS of the “only LEV” and “only VPA” groups did not show significant differences, at 16 months for each. However, the graph in Fig 2D shows a clear difference between the “only LEV” group and the “only VPA” group. The “only both” group and the “only LEV” group showed a similar pattern, and the “only VPA” group had a significantly lower cumulative survival rate than the other two groups. The difference between the numerical results and the graphical interpretation is due to the distinct difference in the censored data in the two groups. As shown in the graph of Fig 2D, the censored data from the “only LEV” group were significantly more than those from the “only VPA” group, which means that the follow-ups for some of the patients in the “only LEV” group were terminated early. In other words, patients who received LEV could survive longer than patients who received VPA by the last follow-up. We found that our cohort showed a tendency to prioritize which AED was used, depending on the time period. According to the analysis of the usage patterns of LEV and VPA in our cohort, the number of VPA prescriptions before 2012 was 5711, which was 76% of the total VPA prescriptions from January 2005 to May 2017, whereas the number of LEV prescriptions before 2012 was 3852, which was only 13% of the total LEV prescriptions during same period. Because data from our cohort were collected until May 2017, the actual OS of patients in the “only LEV” group can be estimated to be longer than the value presented in our results. Thus, it is possible to conclude that the administration of LEV could increase the OS in patients with GBM, compared to the administration of VPA, even when comparing the “only LEV” group with the “only VPA” group.

The analysis of baseline risk factors revealed that a correlation between age and the OS was present (Table 3). To date, several studies have reported that the survival rate in older patients was lower than that in younger patients [2,3,26]. However, this is not a phenomenon restricted to GBM, as the elderly tend to exhibit poorer prognosis and shorter survival with any disease. In addition, Table 3 shows that the administration of LEV and the MGMT promoter status were major factors associated with prolonged survival in patients with GBM. Table 4 analyzes the relationship between major factors based on the results in Table 3, revealing that the administration of LEV positively affected survival in the patient group that had MGMT promoter methylation at diagnosis. Although the exact mechanism for sensitivity to TMZ by LEV is not known, some studies reported on this mechanism. One study demonstrated that LEV inhibits MGMT promoter activity by enhancing the binding of the mSin3A and HDAc1 protein repressor complex to p53 [11]. Another study reported that LEV induces the nuclear translocation of HDAc4 and downregulation of the *MGMT* gene [16]. In other words, LEV further inhibits MGMT expression through a mechanism other than methylation. In GBM patients with a methylated MGMT promoter, the MGMT promoter expression was more firmly inhibited by LEV, thereby increasing the efficacy of alkylating agents such as TMZ. However, the effects of LEV in patients with an unmethylated MGMT promoter were not statistically significant. These findings suggest that the repression of the MGMT promoter caused by methylation has a greater effect on survival than LEV treatment, and LEV can be used as adjunctive therapy in patients with a methylated MGMT promoter. As the number of patients who did not receive LEV among the patients who were tested for the MGMT promoter status was small (Table 4), immediately applying these results to clinical practice will be challenging. Moreover, only a few studies have examined the correlation between LEV treatment and MGMT promoter status and the underlying mechanism associated with the MGMT promoter status remains unclear [11,29]. Hence, both biological and clinical studies on the effects of LEV treatment on the MGMT promoter are warranted.

## Conclusions

This study suggests that LEV administration may prolong survival, compared with other AEDs, in patients with GBM who receive chemotherapy with TMZ. Nevertheless, further randomized control trials are warranted based on recent guidelines related to AED administration in patients with GBM and individual genetic factors associated with GBM should be considered.

## Supporting information

S1 File Clinical data on treatments and survivals. (XLSX)

S2 File Survival analysis data using SPSS program. (ZIP)
