# Correction: Effect of anti-epileptic drugs on the survival of patients with glioblastoma multiforme: A retrospective, single-center study

**DOI:** 10.1371/journal.pone.0228800

**Published:** 2020-02-05

**Authors:** Jae Yeoul Ryu, Kyoung Lok Min, Min Jung Chang

The images for Figs 1 and 2 are incorrectly switched. The image that appears as Fig 1 should be Fig 2, and the image that appears as Fig 2 should be Fig 1. The figure captions appear in the correct order.

**Fig 1:**
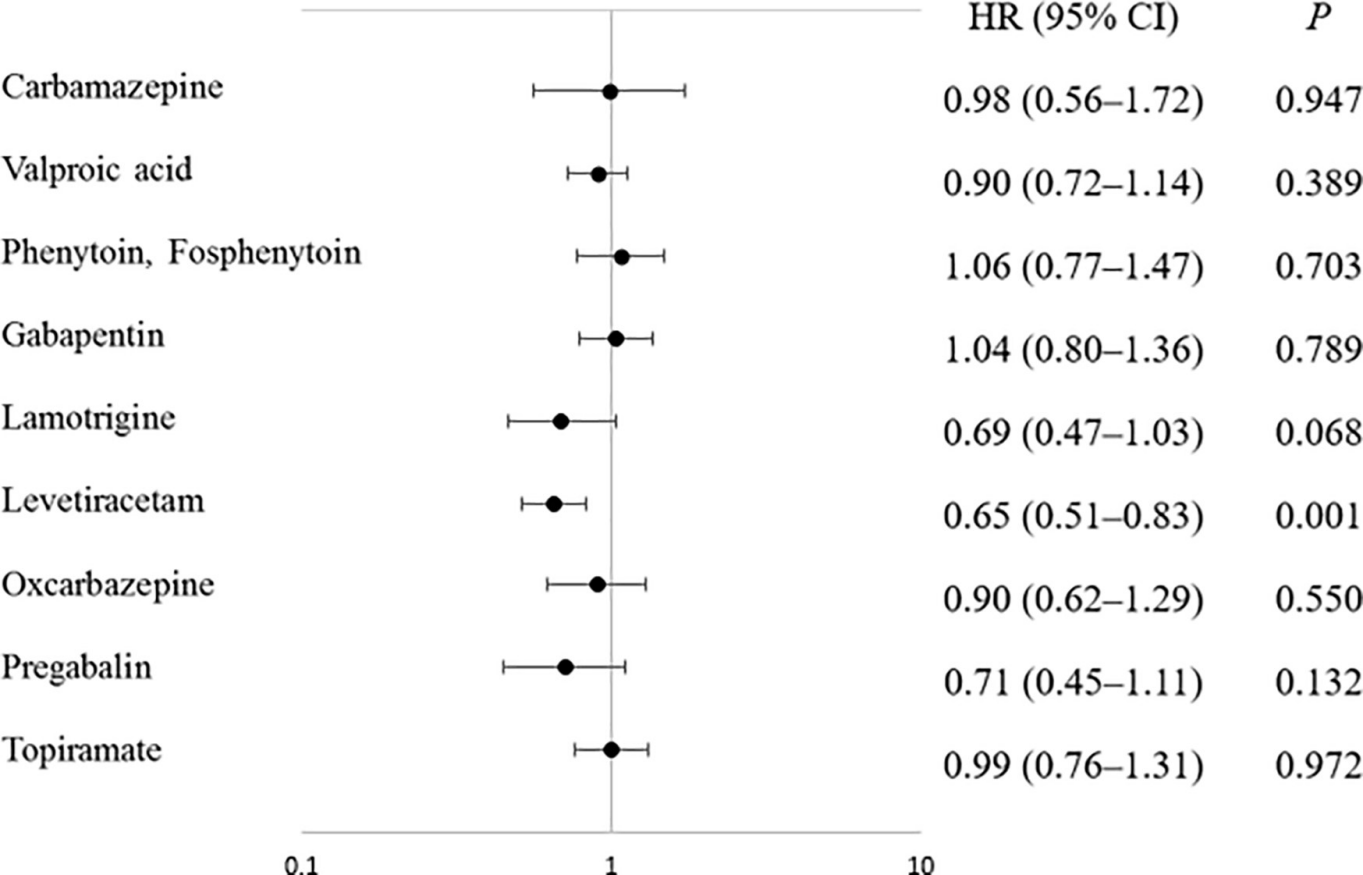
The Cox proportional hazard regression forest plot showing the risk of each antiepileptic drug in patients with glioblastoma. HR, hazard ratio; CI, confidence interval.

**Fig 2:**
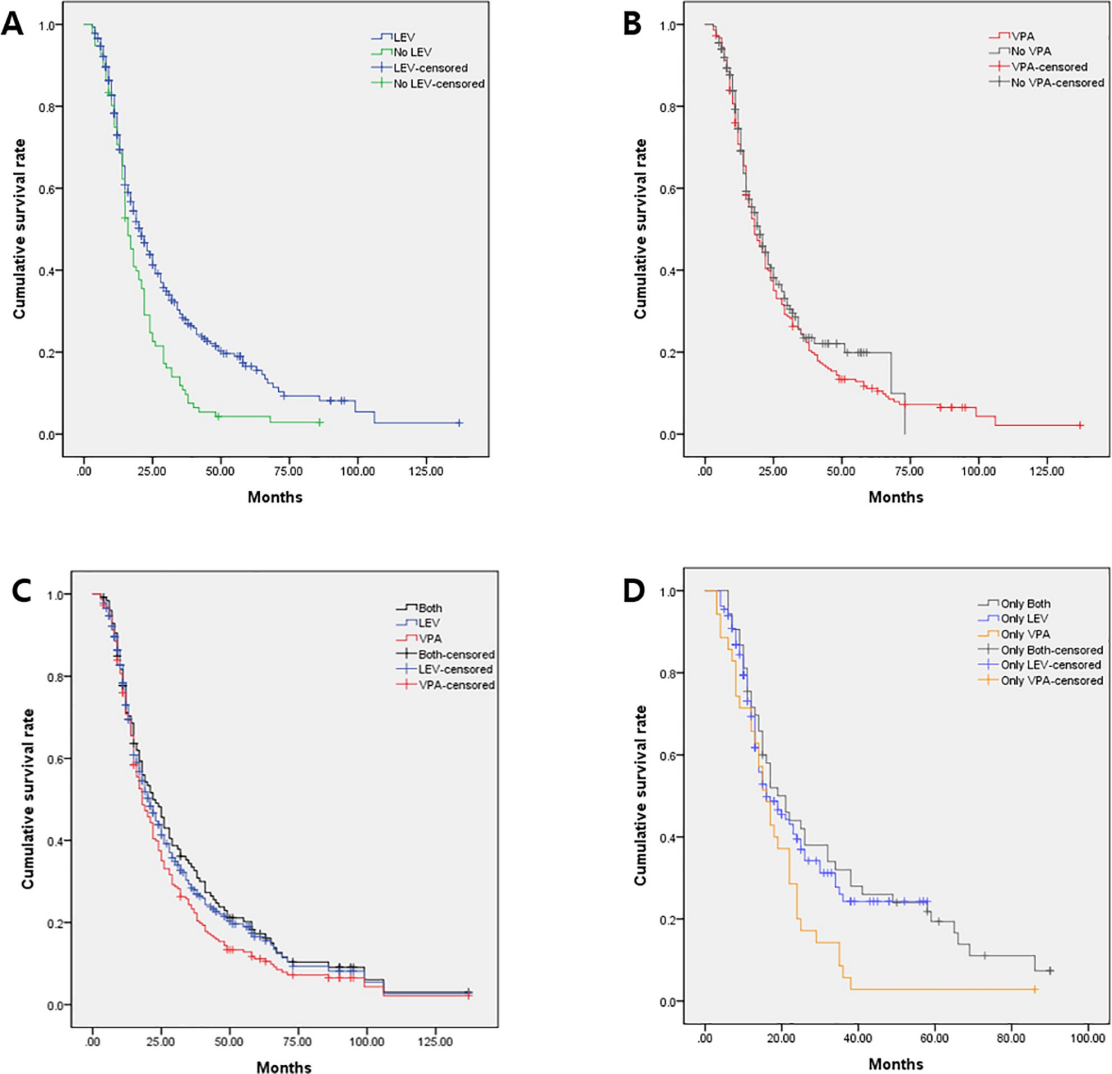
The Kaplan-Meier survival plot showing the overall survival (OS) duration. (a) The LEV-treated group (LEV; blue line) versus no LEV treatment (No LEV; green line). (b) The VPA-treated group (VPA; red line) versus no VPA treatment (No VPA; gray line). (c) The group treated with both LEV and VPA (both; black line) versus the LEV-treated group (blue line) or the VPA-treated group (red line). (d) Treatment with only LEV and VPA (only both; silver line) versus only LEV treatment (only LEV; azure line) or only VPA treatment (only VPA; orange line).
